# Social cognition in aggressive offenders: Impaired empathy, but intact theory of mind

**DOI:** 10.1038/s41598-017-00745-0

**Published:** 2017-04-06

**Authors:** Korina Winter, Stephanie Spengler, Felix Bermpohl, Tania Singer, Philipp Kanske

**Affiliations:** 1 Department of Psychiatry and Psychotherapie, Charité University Medicine Berlin, Berlin, Germany; 2 Department of Forensic Psychiatry, Krankenhaus d. Maßregelvollzugs Berlin, Berlin, Germany; 3 Department of Social Neuroscience, Max Planck Institute for Human Cognitive and Brain Sciences, Leipzig, Germany

## Abstract

Aggressive, violent behaviour is a major burden and challenge for society. It has been linked to deficits in social understanding, but the evidence is inconsistent and the specifics of such deficits are unclear. Here, we investigated affective (empathy) and cognitive (Theory of Mind) routes to understanding other people in aggressive individuals. Twenty-nine men with a history of legally relevant aggressive behaviour (i.e. serious assault) and 32 control participants were tested using a social video task (EmpaToM) that differentiates empathy and Theory of Mind and completed questionnaires on aggression and alexithymia. Aggressive participants showed reduced empathic responses to emotional videos of others’ suffering, which correlated with aggression severity. Theory of Mind performance, in contrast, was intact. A mediation analysis revealed that reduced empathy in aggressive men was mediated by alexithymia. These findings stress the importance of distinguishing between socio-affective and socio-cognitive deficits for understanding aggressive behaviour and thereby contribute to the development of more efficient treatments.

## Introduction

Aggressive behaviour towards others is a severe societal problem. More than 1.1 million cases of violent crimes occur per year in the US alone (such as assault, grievous bodily harm, homicide)^1^. The causes for aggressive behaviour are assumed to be diverse, but deficits in social understanding have been repeatedly proposed as core mechanisms^2, 3^. Nevertheless, evidence for such deficits is limited and, crucially, the specifics of impaired social understanding remain unclear^4^. The present study therefore aims to test if deficits impact affective or cognitive routes of understanding others equally or selectively, thus mainly impairing the ability to share others’ emotions or take others’ perspectives.

Aggressive behaviour is defined as “*any behaviour directed toward another individual that is carried out with the proximate (immediate) intent to cause harm*”^5–8^. It is hypothesised that aggressive behaviour is inhibited when we correctly represent the related consequences for others^9^. This link seems plausible, assuming that those who are neither capable of feeling another person’s pain nor understanding their motives, intentions, and goals are in fact more likely to cross personal boundaries and inflict bodily, psychological or material harm. Deficits in social understanding are also associated with a number of mental disorders and conditions that are characterised by aggressive and violent behaviour, including antisocial personality disorder, psychopathy, and autism spectrum disorder^10–12^. Social psychology and social neuroscience have identified two critical routes to understanding others: an affective route that allows for sharing others’ emotions and feeling for them (including empathy, and compassion) and a cognitive route that enables the representation of and reasoning about others’ mental states (Theory of Mind [ToM], perspective-taking)^13^. While empathy has been defined “*as the process by which an individual infers the affective state of another by generating an isomorphic affective state in the self*, *while retaining knowledge that the cause of the affective state is the other*”^14^, compassion, complementarily, refers to “*an emotional and motivational state characterised by feelings of loving-kindness and a genuine wish for the well-being of others*”^15^. ToM, in contrast, is the ability to understanding others’ mental and affective states by means of reasoning about the thoughts, emotions or beliefs of others^16–18^. This conceptual dissociation is supported by a dissociation of empathy, compassion and ToM on a behavioural and neural basis^19–21^.

In general, aggressive behaviour is not limited to those with mental disorders such as anti-social personality disorder or specific exceptional contexts such as war, but is also carried out by healthy people during everyday social interactions. Regarding aggression in healthy individuals, a couple of meta-analyses^22–24^ have aimed at integrating the literature over the last few years. An early meta-analysis by Miller and Eisenberg^23^ reported a negative association of empathy and aggression in questionnaire studies, but no significant relation for experimental studies, most of which, however, were done in children. Only including questionnaire studies in offenders, Jollife and Farrington^22^ found a weak negative association of aggression with empathy, but a stronger negative relationship to cognitive perspective-taking. More recently, Vachon *et al*.^24^ reviewed all available evidence on social understanding and aggression in adults and also only found a very weak association. Empathy and cognitive perspective taking did not differ in their (non-) relation to aggression. Again, most of the included studies were done using questionnaires. Overall, the evidence is, thus, largely inconsistent. One reason for this may be methodological problems in assessing empathy and Theory of Mind using questionnaires. Both constructs represent socially desirable traits, and questionnaire items assessing them are almost identical to those used in social desirability scales. Also, to allow for direct comparison, empathy and Theory of Mind should be tested in the same individuals. Thus, experimental studies are needed that allow us to assess both empathy and ToM in a group of individuals with aggressive tendencies.

Both empathy and aggression have been related to alexithymia, a personality trait describing difficulties in identifying and expressing one’s own emotional states. The prevalence of alexithymia in the general public is approximately 10%^25^, but it seems to be increased in aggressive individuals^26, 27^. Hitherto, increased alexithymia has been associated with empathy deficits^28–31^ decreased empathic concern, for instance for victims in harmful third-party acts^32^ and for victims in moral dilemmas^33, 34^, and more egocentric moral attitudes^35^, both in healthy adults and clinical groups^36, 37^. There is consistent evidence that people with alexithymia are less empathic as they lack the ability to accurately identify, describe and reflect their own emotions, which makes it even harder—if not impossible—to represent those of others^38^. Recent studies reported that participants with alexithymia showed lower abilities in complex empathy tasks^28, 36, 39–41^. Therefore, aggressive behaviour may be linked to lower empathy capabilities owing to an elevated manifestation of alexithymia.

**Figure 1:**
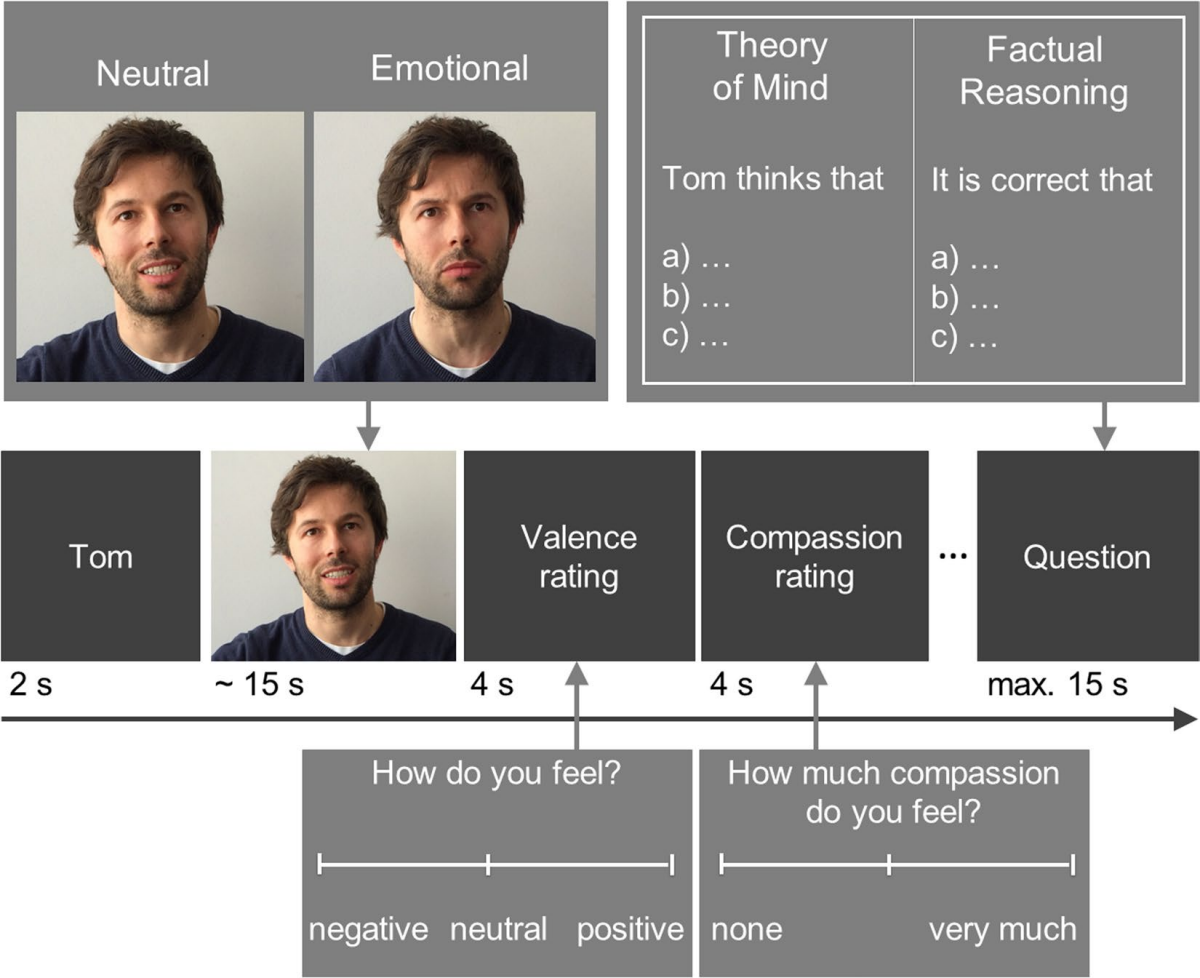
Schematic of the EmpaToM trial sequence and task overview (adapted from Kanske *et al*.^20^).

Taken together, it is yet unclear what the specific deficits that contribute to aggressive behaviour are in social understanding. Therefore, we aimed (1) to test whether social understanding is impaired in men with a history of aggressive behaviour and (2) which components of social understanding are specifically affected. We also hypothesise that (3) a putative deficit in empathy in aggressive healthy men may be mediated by increased alexithymia. To address these questions, we tested empathy, compassion and Theory of Mind in men with a history of legally relevant aggressive behaviour and non-aggressive control participants. We applied a validated experimental paradigm^20^ that presents short video clips of either emotionally negative or neutral valence and asks participants to rate how much they share the narrator’s emotion (empathy rating) and how much compassion they experience for the narrator (compassion rating). Subsequently, multiple-choice questions probed participants’ ToM reasoning and factual reasoning (control condition) capabilities (see Fig. 1). Additionally, we assessed aggression and alexithymia in established questionnaires.

## Results

### Aggression self-reports

As expected, the experimental group showed significantly higher aggression than the control group as indicated in increased Buss-Perry-Aggression-Questionnaire (BPAQ) and Reactive-Proactive Aggression Questionnaire (RPQ) scores (see Table 1, for ﻿repeated analysis with covariates see Supplement S2).

**Table 1:** Characteristics of men with a history of aggressive behaviour and controls, Buss-Perry-Aggression-Questionnaire (BPAQ), Reactive-Proactive Aggression Questionnaire (RPQ) and (Toronto-Alexithymia-Scale-26).

	Aggressive		Controls		*t*	*df*	*p value*	*d (cohen)*
	MEAN	SD	MEAN	SD				
Age	32.172	7.700	31.706	5.713	0.276	61	0.784	−0.068
Years of education	13.966	3.268	17.203	3.108	−3.965	59	0.001^***^	1.012
Intelligence	95.552	11.758	105.438	10.854	−3.415	59	0.001^***^	0.874
*Buss-Perry-Aggression-Questionnaire*								
Physical Aggression	36.759	11.716	21.912	8.346	5.853	61	0.001^***^	−1.460
Verbal Aggression	20.345	5.334	17.618	6.035	1.885	61	0.064	−0.479
Anger	25.690	8.146	17.118	5.493	4.958	61	0.001^***^	−0.382
Hostility	27.483	11.134	18.912	6.820	3.743	61	0.001^***^	−0.928
Aggression Sum Score	110.276	29.366	75.559	20.465	5.505	61	0.001^***^	−1.372
*Reactive-Proactive Aggression Questionnaire*								
Proactive Aggression	5.929	5.956	1.794	2.267	3.737	60	0.001^***^	−0.917
Reactive Aggression	11.339	5.052	4.441	3.751	6.165	60	0.001^***^	−1.550
Reactive-Proactive Aggr. Sum Score	17.268	10.160	6.235	5.549	5.430	60	0.001^***^	−1.348
*Toronto-Alexithymia-Scale*								
Difficulty identifying feelings	14.964	4.484	12.179	3.580	2.569	54	0.013^**^	−0.687
Difficulty describing feelings	14.107	3.178	12.536	4.910	1.422	54	0.161	−0.380
Externally oriented thinking	17.071	4.422	13.857	3.894	2.887	54	0.006^**^	0.772
Alexithymia Sum Score	46.143	6.969	38.571	7.089	4.030	54	0.001^***^	1.077

Note: *Indicates statistical significant p-value: *p ≤ 0.05, **p ≤ 0.01, ***p ≤ 0.001.

### EmpaToM task

#### Empathy ratings

**Figure 2:**
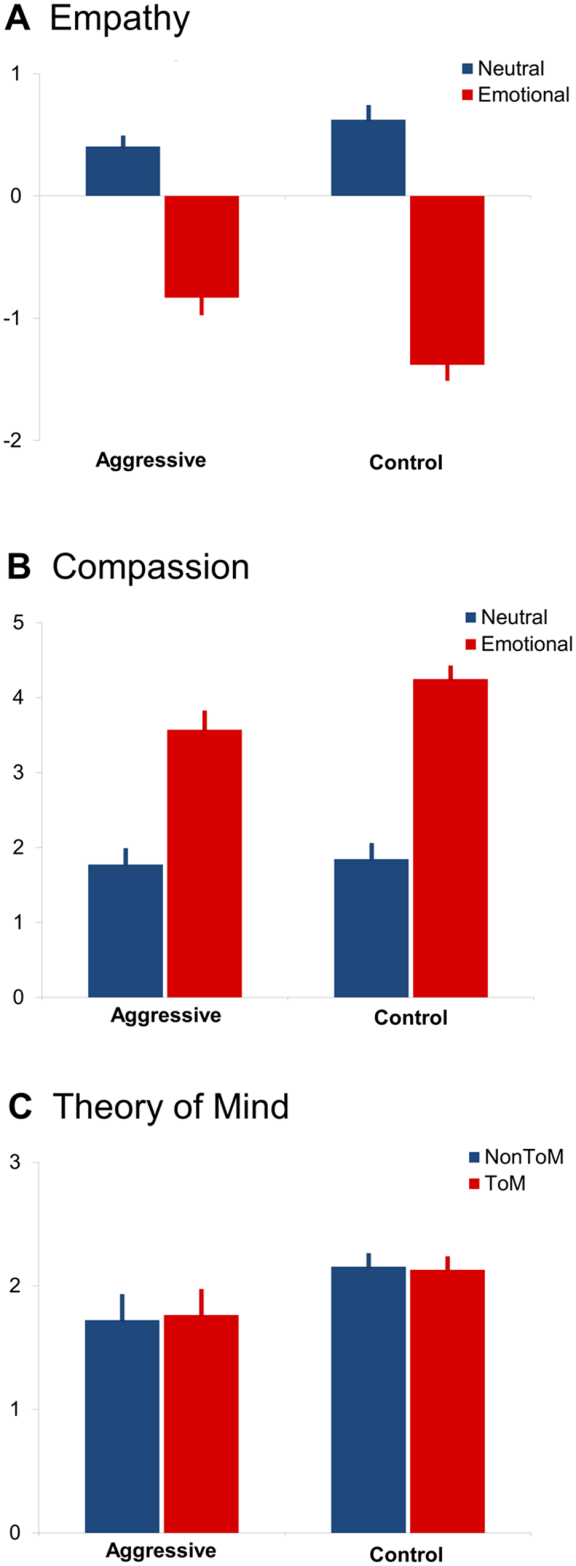
EmpaToM ratings and performance: (**A**) empathy ratings (emotional and neutral condition), (**B**) compassion ratings (emotional and neutral condition), and (**C**) ToM and factual reasoning performance (composite score of error rates and reaction time ratings, performance scores were z-transformed and for display purpose depicted with a mean of 2) for the aggressive and the control group. Error bars represent 95% confidence intervals.

Regarding the main effect of valence, all participants showed more negative affect ratings for emotionally negative videos than neutral videos (see Fig. 2A and Table 2; *F*_(1,57)_ = 140.31, *p* < *0.001*, η² = 0.711, d = 3.16). There was no significant main effect of *group* (*F*_(1,57)_ = 7.91, *p* = 0.152, η² = 0.122, d = 0.075). Most importantly, we found a significant interaction of *group* and *valence* (*F*_(1,57)_ = 7.910, *p* = 0.007, η² = 0.122, d = 0.746). Post-hoc analysis revealed that the experimental group showed significantly less negative affect after watching emotionally negative videos than the control group (mean difference = −1.107, SD = 0.097, F_(1,57)_ = 8.05, p = 0.001, η² = 0.124, d = 0.75). This reduced sharing of negative affect indicates diminished empathy in the aggressive group. After including IQ and years of education as covariates, the significant interaction effect remained significant (see Supplement S4).

**Table 2:** EmpaToM measures analysed by means of separate repeated-measures analyses of variance.

*EmpaToM behaviour*		*F*	*df*	p value	*eta²*	*d*
*Empathy*						
Main Effect of Group		7.91	57	0.152	0.122	0.075
Main Effect of Valence		140.31	57	0.001***	0.711	3.16
Interaction		7.910	57	0.007**	0.122	0.746
Post-hoc	MeanDiff = −1.107	8.05	57	0.001***	0.124	0.75
*Compassion*						
Main Effect of Group		2.119	57	0.151	0.036	0.388
Main Effect of Valence		155.50	57	0.001***	0.732	3.327
Interaction		3.21	57	0.079	0.53	0.47
Post-hoc	MeanDiff = 3.911	4.93	57	0.030*	0.080	0.59
*ToM*						
Main Effect of Group		3.55	57	0.065	0.060	0.506
Main Effect of ToM		0.499	57	0.483	0.0089	0.019
Interaction		0.87	57	0.356	0.016	0.251

Note: *Indicates statistical significant p-value: *p ≤ 0.05, **p ≤ 0.01, ***p ≤ 0.001.

#### Compassion ratings

All participants reported more compassion after watching emotionally negative videos compared to neutral videos (see Fig. 2B, and Table 2; F_(1,57)_ = 155.50, p < 0.001, η² = 0.732, d = 3.327). There was no significant main effect of *group* (F_(1,57)_ = 2.119 p = 0.151, η² = 0.036, d = 0.388). The interaction of *emotionality* and *group* was marginally significant (F_(1,57)_ = 3.21, p = 0.079, η² = 0.53, d = 0.47), pointing to reduced compassion after emotionally negative videos in the experimental group compared to the control group (mean difference = 3.911, SD = 0.152, F_(1,57)_ = 4.93, p = 0.030, η² = 0.080, d = 0.59). This effect remained marginally significant after including IQ as covariate, the main effect of valence remained significant as well (see Supplement S4).

#### Theory of Mind performance

The main effect of ToM performance vs. factual reasoning was not significant in the entire sample: Performance was the same for the ToM and factual reasoning questions (see Fig. 2C and Table 2; *F*_(*1*,*57*)_ = 0.499, p = 0.483, η² = 0.0089, d = 0.019). The main effect of *group* was marginally significant (F_(1,57)_ = 3.55, p = 0.065, η² = 0.060, d = 0.506). Critically, the interaction between *group* and *ToM* was not significant (F _(1,57)_ = 0.87, p = 0.356, η² = 0.016, d = 0.251). Including IQ as covariate did not change this pattern, but led to a further, strong reduction of the size of the group main effect (see Supplement S4).

### Relations of aggression with EmpaToM behaviour

Corroborating the findings, RPQ scores correlated negatively across groups with empathy ratings (see Supplement S4 and S5; RPQ: r = −0.342, p = 0.004; BPAQ: r = −3.22, p = 0.048 n.s. after Bonferroni correction) and compassion ratings (see Supplement S4 and S5; RPQ: r = −0.355, p = 0.006, BPAQ: r = −0.132, p = 0.319 n.s.), but not with ToM performance (BPAQ: r = 0.072, p = 0.589; RPQ: r = 0.089, p = 0.505).

### Relations of alexithymia with aggression and EmpaToM behaviour

#### Alexithymia and Aggression

Participants with a history of aggressive behaviour reported significantly higher scores of alexithymia than controls (see Table 1). Across groups, alexithymia was correlated with aggression as measured in the BPAQ (see Supplement S3 and S6; r = 0.37, p = 0.005) and the RPQ (r_s_ = 0.32, p = 0.017).

#### Alexithymia and EmpaToM measures

Empathy and compassion correlated negatively across groups with alexithymia (see Supplement S3 and S6; empathy: r = −0.35, p = 0.001, compassion: r_s_ = −0.36, p = 0.016). There was no significant correlation between ToM performance and alexithymia (r = 0.137, p = 0.332). Interestingly, compassion and empathy scores correlated negatively with the TAS subscale “externally oriented thinking” (empathy ratings: r = −0.374, p = 0.006; compassion ratings: r = −0.525, p < 0.001), but not with ToM scores after Bonferroni correction (r = −0.247, p = 0.078).

### Pathmodel of alexithymia in aggression

**Figure 3:**
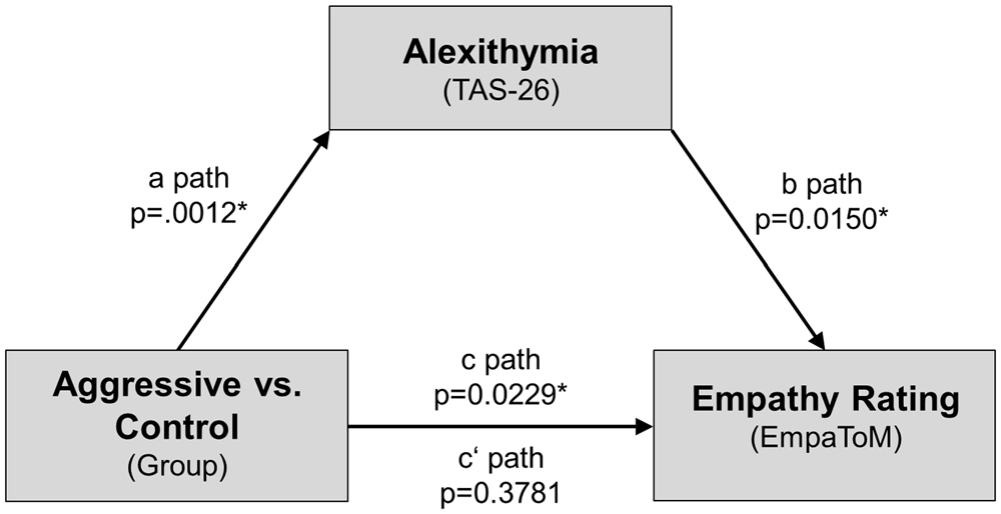
Mediation model for the effect of aggressive behaviour on empathic responses, alexithymia is modelled as mediator.

Mediation analysis^42^ was used to test the hypothesis that the empathy deficit in aggressive participants is mediated by increased alexithymia (see Fig. 3). This analysis revealed a significant relationship between group (men with a history of aggressive behaviour vs. controls) and empathy ratings (c path: coeff = 0.4988, se = 0.2126, t = 2.3457, p = 0.0229). This relationship was statistically not significant when alexithymia was included as mediator (c’ path: effect = 0.2248, se = 0.2528, t = 8892, p = 0.3781). Furthermore, the mediator variable (alexithymia scores) was associated with both group (coeff = −6.4686, se = 1.8870, t = −3.4279, p = 0.0012) and empathy ratings (coeff = −0.0424, se = 0.0168, t = −2.5193, p = 0.0150). Bootstrapping procedures were used to test the significance of the mediation effect. The bias-corrected bootstrap confidence interval for the indirect effect (c path) based on 1000 bootstrap samples was above 0 (0.0774–0.6125). Additionally, a partial correlation analysis was conducted between group affiliation, empathy ratings, and alexithymia scores to validate these results. As expected, no significant correlation was found (r_s_ = 0.145, p = 0.306). In conclusion, the mediation analysis revealed alexithymia as crucial mediator for lower empathy ratings in men with a history of aggressive behaviour. Given that groups differed with regard to verbal IQ and years of education, mediation analyses were repeated using these variables as covariates (see Supplement S7), which altered the results.

To further assess the influence of verbal IQ and years of education, within-group correlation analyses were carried out between both empathy and compassion measures and verbal IQ, as well as between both empathy and compassion measures and years of education (see Supplement S8). These analyses did not show an association between empathy and compassion measures on the one hand and measures of intelligence and education on the other. Although the interaction effect for the compassion ratings in the EmpaToM task was only marginally significant, an exploratory mediation analysis was conducted, which showed that alexithymia does not mediate reduced compassion in the experimental vs. control group (see Supplement S7).

## Discussion

The present study yields several new insights into the role of social understanding and alexithymia for aggressive behaviour. Firstly, men with a history of aggressive behaviour showed decreased sharing of negative affect with others, indicating diminished empathy, and reduced compassion after emotionally negative videos. Secondly, no ToM deficit was found, demonstrating intact cognitive perspective-taking in men with a history of aggressive behaviour. These results were observed both when comparing men with and without a history of aggressive behaviour and when correlating aggression severity with empathy, compassion and ToM. Thirdly, the empathy deficit in men with a history of aggressive behaviour was mediated by increased alexithymia.

The results of the present study confirm the hypothesised link between aggression and impaired social understanding, showing that it is a deficit in feeling another person’s pain, but not the reasoning about their motives, intentions, and goals, that allows crossing personal boundaries and inflicting bodily harm. Thus, the present data clarify the on-going questions of whether and how aggression relates to impaired social understanding. While previous meta-analyses yielded largely inconsistent evidence^22, 24^, the clear results observed here speak for an experimental operationalization of social understanding, which, in contrast to the mostly applied questionnaire assessments, is not subject to response tendencies, for example related to social desirability. It also seems critical to test the different aspects of social understanding within the same individuals to allow conclusions about their specific impairment. Lastly, replicating group differences with correlational data validates the observed relationships as suggested by Vachon *et al*.^24^ and by Mariano *et al*.^43^.

The observed deficit in healthy men with a history of aggressive behaviour in affective social understanding is in line with evidence from psychopathology. For instance, psychopathy, which is characterised by severe antisocial behaviour, has been related to reduced empathic responses, while Theory of Mind performance is not affected^44–49^. A similar pattern has been reported in patients with narcissistic personality disorder, who also show reduced prosocial behaviour^50, 51^. A primary lack of empathy and compassion is also found in frontotemporal dementia and, more generally, the frontal lobe syndrome. Paralleling our present findings, studies of these conditions reported a lack of empathy and compassion associated with elevated levels of aggressive behaviour^52–56^.

Violent behaviour toward others and themselves has also been reported in some patients with autism spectrum disorder^57, 58^, which is mainly associated with deficits in ToM, but not empathy^37, 59, 60^. However, prevalence for alexithymia is largely increased in autism and can lead to reduced empathic responses in autistic individuals as well^31^, thus raising the question of whether the aggressive behaviour is primarily associated with the ToM deficits or rather alexithymia^37^. The present findings highlight that the relationship between lack of empathy and aggressive behaviour is not confined to people with psychiatric disorders but is also crucial in understanding aggression in healthy individuals, such as criminal offenders.

In line with our findings, recent studies reported that criminal offenders had no deficits in judging other people’s behaviour as right or wrong – possibly indicating intact ToM – but had deficits in sharing the suffering of other people^43^, as well as deficits in emotion recognition and empathy in ecological, context-sensitive measures^61^.

Our findings are also in line with investigations that link enhanced social understanding to prosocial behaviour^4, 23, 62, 63, 64^ possibly through enhanced early detection of others’ emotions^13, 20^. While the specific and relative contributions of empathy, compassion and ToM to prosocial behaviour are not yet entirely clear, some first evidence demonstrated that training in compassion-focused meditation can increase helping and non-selfish behaviour in interactive game paradigms^65, 66^. This may be an avenue for future intervention studies in chronically aggressive individuals.

Alexithymia has already been shown to play an important role in empathic responses, which was replicated in this study^28, 36, 38, 39, 67^. In General, alexithymia, as the ability to empathise with other people’s emotional states relies on parts of those networks that are involved when the emotional states are experienced by oneself, difficulties in identifying one’s own feelings seem to be paralleled by reduced empathy^31^. The present findings suggest that the empathy deficit in men with a history of aggressive behaviour is mediated by increased alexithymia, suggesting that it may actually be aberrant alexithymia that brings about reduced empathic responses. Further suggestions for the relation of alexithymia to aggression have been made by Zillmann^68^, assuming that monitoring one’s own level of excitement is crucial for leaving dangerous situations, which people with high levels of alexithymia may consequently be unable to do^69^. Furthermore, awareness of one’s own emotions is correlated with tolerating negative emotions^70^, and a reduced capacity to identify emotions results in more maladaptive coping-styles^71, 72^. Training emotional awareness may, therefore, be another promising approach in psychotherapeutic intervention.

There are some limitations to the present study. On average the group of men with history of aggressive behaviour scored lower on all measures of education and intelligence. When years of education and verbal IQ were used as covariates in our analyses, the results of the group-by-valence interaction and the group difference in the negative valence condition remained largely the same, while the mediating effect of alexithymia on empathic responding did not remain significant. It is thus acknowledged that we cannot fully exclude that differences in education and IQ may play a role and that future studies in groups matched for education and IQ will have to replicate the present findings. In line with conceptual considerations, the present data did, however, not reveal a correlation between empathy measures and years of education or IQ nor between compassion measures and years of education or IQ, when the two groups were analysed separately (see Supplement S8). Thus, empathy and compassion and the deficits therein observed in the group of men with a history of aggressive behaviour seem not to be linked with education and intelligence. In contrast, one may rather have expected differences in education and IQ to affect performance in ToM^73^. Strikingly, we found no group difference in ToM measures, suggesting again that, in the present study, group differences do not simply and unspecifically occur due to differences in education and intelligence^74^.

A second point concerns the ratings in the EmpaToM, which are still subjective in nature. However, the EmpaToM ratings have previously been shown to directly trace neural responses in empathy related brain regions on a trial-wise level and also correspond to changes in heart rate^20^. Future studies in aggression should, nevertheless, include more of such objective measures, in particular to elucidate the influence of differences in alexithymia on subjective and objective affective social understanding. Despite the exclusion of DSM-IV Axis I and II disorders, psychopathy^67^ and other inter-individual difference characteristics such as cognitive schemata and scripts^75, 76^ were not assessed. Future research should include such measures to test the specific relations to aggressive behaviour. Lastly, we only tested aggression in men. While the prevalence for physical aggression, in particular, is much lower in women^77^, it still remains to be tested whether the same mechanisms observed here can be generalised to women.

To conclude, based on the finding that affective and cognitive routes to understanding others are distinct and can be assessed separately^20^, we investigated the role that deficits in these social functions play for aggressive behaviour. We observed a selective deficit in affective responses, but not cognitive perspective-taking, in men with a history of aggressive behaviour, which suggests that it is the sharing of others’ emotions and feeling for them, that inhibit aggression. Deficits in cognitive understanding of others’ mental states, however, do not play a critical role for aggressive behaviour. Selectivity of the impairment also corroborates the separation of affective and cognitive routes to social understanding. Furthermore, as alexithymia mediates reduced empathic responses in men with a history of aggressive behaviour, the present results underline the importance of awareness of one’s own emotions for affect sharing and allow suggestions for future developments in psychotherapeutic treatment to include emotional awareness training.

## Methods

### Participants

A total sample of N = 63 participants (all male, all without mental disorders) was recruited via flyers that were posted in online communities and via focus groups that were directly contacted. With N = 29 in the aggressive group (participants with a history of aggressive behaviour) and N = 34 in the control group. Participants with history of aggression were included if they had committed at least one criminal act of assault or grievous bodily harm with deceitful intent, such as attempted homicide, knife assaults causing bone fractures or injuries of vital organs, or physical assault resulting in brain bleeding and fractures at the base of the skull^78^ (note that this did not always result in incarceration, but could also be punished with community service or psychological treatment). All participants were screened by a clinical psychologist to exclude any Axis I or II disorder and drug abuse within the last 6 months using the Structured Clinical Interview for DSM-IV I & II^79^. There were no significant age differences between the two groups (see Table 1), but significant differences between school leaving qualifications, and length of formal education. The Wortschatztest (WST), a German vocabulary and speech comprehension test, was used to evaluate verbal intelligence^80^. The test﻿ is a German equivalent of the National Adult Reading Test (NART)^81^. The sum scores of the WST were converted to IQ scores, which also differed significantly between the groups, possibly because the members of the aggressive group left formal education earlier.

The methods of the present study adhere to the Declaration of Helsinki (1964) and all experimental protocols were approved by the ethics committee of the Charité – Universitätsmedizin Berlin (number EA1/119/13). All participants gave written informed consent prior to participation.

### Questionnaires

Differences in aggressive behaviour between the groups were assessed using:

1. The *Buss-Perry-Aggression-Questionnaire* (BPAQ, Buss and Perry^82^), which is one of the most widely used self-report tools to assess aggression. The German revised version was used^83^ for the present study^84^. The BPAQ consists of 29 items forming four subscales (physical and verbal aggression, anger and hostility). The sum score is used as an indicator of overall “dispositional aggression”.
2. The *Reactive-Proactive Aggression Questionnaire* (RPQ, Raine *et al*.^85^), which assesses reactive aggression (RA) and proactive-aggression (PA) and are added up to a sum score. This consists of 23 items, 11 measuring RA, 12 PA on a 3-point-scale.

Additionally, the *Toronto- Alexithymia-Scale-26* (TAS-26, Taylor *et al*.^25^; rev. German version Kupfer *et al*.^86^) was assessed to test for differences in alexithymia. It consists of 18 Items forming 3 subscales (difficulty identifying feelings, difficulty describing feelings, externally oriented thinking). The sum score is used as an indicator of overall alexithymia.

### Task

The EmpaToM task^20^ was employed to assess empathy, compassion and ToM (see Fig. 1). In a series of neuroimaging studies, these different measures of the EmpaToM have been validated with external tasks and self-reports. The EmpaToM consists of 48 short video (~15 s) sequences that differ in emotional valence (negative vs. neutral). Videos are followed by two rating questions (each 4 s), asking for (1) the valence of the current emotional state (negative – neutral – positive, empathy measure) and (2) the level of compassion felt for the person in the video (none – very much, compassion measure). Participants were presented with sliding rating scales without numbers, but for analysis, the responses in the ratings were coded on a scale from −3 (negative) to +3 (positive). A subsequent multiple-choice question (max. 15 s) either demanded a ToM inference or factual reasoning (control condition) on the content of the previous video (ToM performance measure). Lastly, a confidence rating (4 s) probes meta-cognition by asking participants how confident they were when choosing the correct response in the previous question. We excluded the latter aspect from the present analysis because it was not the main focus of the current research aims.

### Data analysis

All statistical analyses were performed using IBM^®^ SPSS^®^ Statistics Version 22. Effect sizes are based on Cohen^87^ and Rosenthal^88^. Normal distribution was tested using the Shapiro-Wilk-Test (see Supplement S1) and the used variables did not significantly deviate from a normal distribution (all p > 0.05). Therefore, parametric tests were used for the statistical analysis (ANOVA, Pearson’s product-moment-correlation).

The ratings (affect, compassion) and performance measure of ToM (error rates and reaction times in the questions were combined into one z-transformed and averaged composite score (for each condition), see Kanske *et al*.^20^) were analysed by means of separate repeated-measures analyses of variance (ANOVAs). A 2 × 2 factorial design was applied with the within-subject factors Emotionality of Video (emotionally negative vs. neutral videos) and a between-subject factor group (aggressive vs. control participants) to analyse empathy and compassion ratings. For simplification the empathy-scoring scheme was multiplied by minus one so that high scores in the rating system reflect high empathic responding. ToM performance was analysed with a 2 × 2 factorial design with the within-subject factors ToM (ToM versus factual reasoning) and the between-subject factor group (aggressive vs. control participants). Significance was set at p < 0.05. Post hoc analyses were performed using a univariate ANOVA with Bonferroni correction for multiple comparisons (p = 0.05/3 = 0.017).

To test for associations with aggression and alexithymia questionnaires, the EmpaToM measures were correlated with the BPAQ, RPQ and TAS. Bonferroni correction was applied to account for multiple testing (p ≤ 0.017). Lastly, a mediation analysis was calculated to elucidate whether alexithymia mediates a putative empathy deficit in men with a history of aggressive behaviour. As participants could not be matched for intelligence and years of education we repeated all analyses including IQ and years of education as covariates to confirm that the results were not influenced by different IQ scores or years of education.

## Electronic supplementary material

Supplement
