# Supplementary material for: Social cognition in aggressive offenders: Impaired empathy, but intact theory of mind
Source: Sci Rep. 2017 Apr 6;7:670. doi: 10.1038/s41598-017-00745-0 (PMC5429629; doi:10.1038/s41598-017-00745-0)
Supplement: Supplementary file 1 — Supplement [file 41598_2017_745_MOESM1_ESM.pdf]

Supplementary material

Social cognition in aggressive offenders:

Impaired empathy, but intact theory of mind

Korina Winter[1,2], Stephanie Spengler[1], Felix Bermpohl[1], Tania Singer[3], Philipp Kanske[3*]

[1]Department of Psychiatry and Psychotherapie, Charité-Universitätsmedizin Berlin, Germany

[2]Department of Forensic Psychiatry, Krankenhaus d. Maßregelvollzugs Berlin, Germany

[3]Department of Social Neuroscience, Max Planck Institute for Human Cognitive and Brain Sciences, Leipzig, Germany

*Corresponding author:

Philipp Kanske, Department of Social Neuroscience, Max Planck Institute for Human Cognitive and Brain Sciences, Stephanstr. 1a, 04103 Leipzig, Germany (e-mail: kanske@cbs.mpg.de)

S1 Normal Distribution Testing: *Demonstrating used variables did not significantly deviate from normal distribution (all p> .05), parametric tests were used for the statistical analysis.*

Questionnaire/Scales	***Shapiro-Wilk***	***asymmetry***	***kurtosis***
Aggression (BPAQ sum score)	.105	.340	-.710
Physical Aggression (BPAQ)	.034	.381	-.627
Anger (BPAQ)	.040	.422	-.648
Hostility (BPAQ)	.027	.660	.079
Verbal Aggression (BPAQ)	.602	.129	-.324
Reactive Aggression (RPQ)	.009	.514	-.489
Proactive Aggression (RPQ)	.000	1.852	3.035
Alexithymia (TAS sum score)	.560	-.324	-.044
Difficulty identifying feelings (TAS)	.090	.414	-.371
Difficulty describing feelings (TAS)	.107	-.262	-.412
Externally oriented thinking (TAS)	.141	.475	-.047
Intelligence (Wortschatz WST)	.055	-.451	-.139
Empathy rating emotional	.970	.90	-.754
Emapthy rating neutral	.920	1.065	1.343
Compassion rating emotional	.889	-1.384	2.170
Compassion rating neutral	.914	-.159	-1.454
ToM performance	.0571	-.77	0,388
Factual reasoning performance	.0388	-.506	1,051

Note: *BPAQ Buss-Perry-Aggression-Questionnaire, RPQ=Reactive-Proactive-Aggression, TAS= Toronto-Alexithymia-Scale, Intelligence=Verbal Intelligence/Wortschatztest.*

S2 Group Differences in aggression and alexithymia self-reports: *Univariate analysis with IQ and years of education as covariate reporting group differences between control and experimental (=men with a history of aggressive behaviour) groups, demonstrating significantly higher aggression in the experimental compared to the control group (as indicated in increased Buss-Perry-Aggression-Questionnaire (BPAQ) and Reactive-Proactive Aggression Questionnaire (RPQ) scores), as well as higher scores of alexithymia (Toronto-Alexithymia-Scale).*

	Aggressive		***Controls***		***Covariates***					
					IQ			***Years of education***		
	MEAN	***SD***	***MEAN***	***SD***	***F***	***p value***	***d (cohen)***	***F***	***p value***	***d (cohen)***
Buss-Perry-Aggression-Questionnaire										
Physical Aggression	36.759	11.716	21.912	8.346	6.027	.017**	0.655	29.228	.001***	1.5
Verbal Aggression	20.345	5.334	17.618	6.035	.150	.700	0.103	2.116	.152	.402
Anger	25.690	8.146	17.118	5.493	3.939	.052	0.529	19.454	.001***	1.22
Hostility	27.483	11.134	18.912	6.820	4.100	.047*	0.54	8.684	.005**	.143
Aggression Sum Score	110.276	29.366	75.559	20.465	5.272	.025*	0.613	21.754	.001***	.817
Reactive-Proactive Aggression Questionnaire										
Proactive Aggression	5.929	5.956	1.794	2.267	5.630	.021**	0.633	7.613	.008**	.766
Reactive Aggression	11.339	5.052	4.441	3.751	2.128	.150	0.389	34.399	.001***	1.62
Reactive-Proactiv Aggr. Sum Score	17.268	10.160	6.235	5.549	4.068	.048**	0.538	21.673	.001***	1.29
Toronto-Alexithymia-Scale										
Difficulty identifying feelings	14.964	4.484	12.179	3.580	.958	.332	0.261	4.976	.030**	.617
Difficulty describing feelings	14.107	3.178	12.536	4.910	1.307	.258	0.305	1.019	.317	.278
Externally oriented thinking	17.071	4.422	13.857	3.894	5.902	.018**	0.648	4.382	.078	.581
Alexithymia Sum Score	46.143	6.969	38.571	7.089	6.457	.014**	0.678	9.985	.003**	.876

*Note: *indicates statistical significant p-value:* $* p \leq 0.05$, $** p \leq 0.01$, $*** p \leq 0.001$

S3 Correlations between EmpaToM ratings and aggression/alexithymia questionnaires applying IQ & years of education as covariates:

S3 (A) Bivariate correlations with IQ as covariate endorse significant negative correlation between compassion and aggression (reactive-proactive-aggression; RPQ).

	Buss-Perry-Aggression-Qu. (BPAQ; Sum Score)		***Reactive-Proactive-Aggression (RPQ, Sum Score)***		***Toronto Alexithymia Scale (TAS-26, Sum Score)***	
	r	*p value*	*r*	*p value*	*r*	*p value*
EmpaToM behaviour						
Empathy	-.283	.044	-.322	.021	-.268	.058
Compassion	-.135	.345	-.369	.008**	-.312	.026
ToM	.228	.108	.084	.559	.141	.324

	Toronto Alexithymia Scale (TAS-26, Sum Score)	
	r	*p value*
Questionnaires		
BPAQ Sum Score	.229	.107
RPQ Sum Score	.272	.157

*Note: Bonferroni correction was applied to account for multiple testing *p≤.017*

S3 (B) Bivariate correlations with years of education as covariate indicating a negative correlation between both empathy and compassion and aggression (RPQ) as well as a negative correlation between empathy and alexithymia.

	Buss-Perry-Aggression-Qu. (BPAQ; Sum Score)		***Reactive-Proactive-Aggression (RPQ Sum Score)***		***Toronto Alexithymia Scale (TAS-26, Sum Score)***	
	r	*p value*	*r*	*p value*	*r*	*p value*
EmpaToM behaviour						
Empathy	-.208	.140	-.347	.012**	-.402	.003**
Compassion	-.132	.351	-.388	.004**	-.309	.026
ToM	.210	.138	-.085	.554	.104	.467

	Toronto Alexithymia Scale (TAS-26, Sum Score)	
	r	*p value*
Questionnaires		
BPAQ Sum Score	.245	.080
RPQ Sum Score	.212	.131

*Note: Bonferroni correction was applied to account for multiple testing *p≤.017*

S4 EmpaToM measures analysed by means of separate repeated-measures analyses of variance with IQ and years of education as covariate:

S4(A) EmpaToM behaviour with IQ as covariate: significant interaction effect between group and valence in the empathy condition. Post-hoc analysis with IQ as covariate approves likewise that the experimental group showed significantly less negative affect after watching emotionally negative videos (=emotional negative condition) than the control group. In the compassion condition with IQ as covariate main effect of valence remained significant, as well as the marginally significant interaction of emotionality and group.

EmpaToM behaviour	*F*	*df*	*Sig.*	*eta²*	*d*
Empathy. ANCOVA covariate intelligence (WST)					
Main Effect of Group	2.001	1.56	.163	.1467	.383
Main Effect of Valence	2.109	1.56	.152	.0372	.393
Interaction (group vs. emotionality)	4.478	1.56	.039*	.077	.577
Post-hoc: Emotional negative condition vs. Group					
Mean Diff.= -1.102 (SE=.101)	5.271	1.56	.026*	.0882	.622
Compassion. ANCOVA covariate intelligence (WST)					
Main Effect of Group	1.817	1.56	.183	.0322	.365
Main Effect of Valence	9.916	1.56	.003**	.1536	.852
Interaction (group vs. emotionality)	3.055	1.56	.086	.054	.478
Post-hoc: Emotional negative condition vs. Group					
Mean Diff.= 3.902 (SE=.158)	4.294	1.56	.043**	.074	.530
ToM. ANCOVA covariate intelligence (WST)					
Main Effect of Group	.753	1.56	.389	.0136	.235
Main Effect of ToM	1.974	1.56	.166	.0354	.383
Interaction	.274	1.56	.603	.005	.142

*Note: *indicates statistical significant p-value:* $* p \leq 0.05$, $** p \leq 0.01$, $*** p \leq 0.001$

S4(B) EmpaToM behaviour with years of education as covariate: interaction effect between group and valence remained significant in the empathy condition. The subsequent post-hoc analysis with years of education as covariate confirmed that the experimental group showed significantly less negative affect after watching emotionally negative videos (=emotional negative condition) than the control group. In the compassion condition either the main effect of valence nor the interaction effect did remained (marginally) significant.

EmpaToM behaviour	*F*	*df*	*Sig.*	*eta²*	*d*
Empathy. ANCOVA covariate years of education					
Main Effect of Group	1.963	1.56	.167	.0338	.374
Main Effect of Valence	1.668	1.56	.202	.0289	.345
Interaction (group vs. emotionality)	5.787	1.56	.019**	.0934	.642
Post-hoc: Emotional negative condition vs. Group					
Mean Diff.: -1.109 (SE=.097)	6.313	1.56	.015**	.101	.695
Compassion. ANCOVA covariate years of education					
Main Effect of Group	2.038	1.56	.159	.035	.381
Main Effect of Valence	.095	1.56	.760	.0017	.082
Interaction (group vs. emotionality)	2.687	1.56	.107	.0456	.437
Post-hoc: Emotional negative condition vs. Group					
Mean Diff.: 3.911 (SE=.153)	4.463	1.56	.039**	.074	.546
ToM. ANCOVA covariate years of education					
Main Effect of Group	.460	1.56	.501	.0082	.182
Main Effect of ToM	.491	1.56	.918	.0088	.188
Interaction (Group vs. ToM)	.500	1.56	.483	.0089	.19

*Note: *indicates statistical significant p-value:* $* p \leq 0.05$, $** p \leq 0.01$, $*** p \leq 0.001$

S5 Correlation between EmpaToM measures and aggression self-reports*: Reactive-Proactive-Aggression scores correlated negatively across groups with empathy ratings (RPQ: r=-.342, p=.004; BPAQ: r=-3.22, p=.048 n.s. after Bonferroni correction) and compassion ratings (RPQ: r=-.355, p=.006; BPAQ: r=-.132 p=.319), but not with ToM performance (BPAQ: r=.072, p=.589; RPQ: r=.089, p=.505). For simplification the empathy-scoring scheme was multiplied by minus one so that high scores in the rating system reflect high empathic responding.*

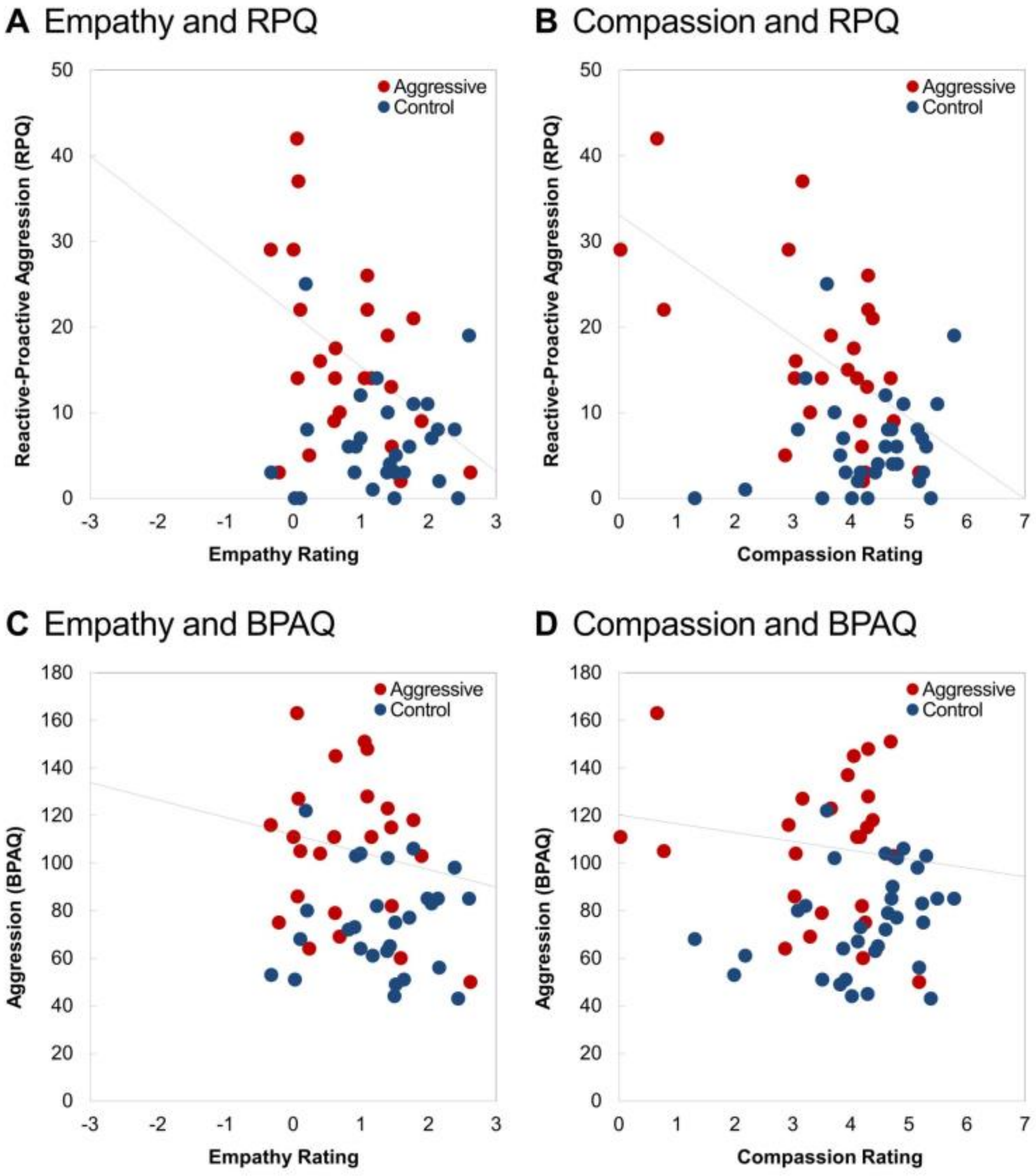

S6 Correlation between Alexithymia and EmpaToM measures/ aggression self-reports: *Empathy and compassion correlated negatively across groups with alexithymia (empathy: r=-.460, p=.001, compassion: r=-.32, p=.016). For simplification the empathy-scoring scheme multiplied by minus one so that high scores in the rating system reflect high empathic responding*.

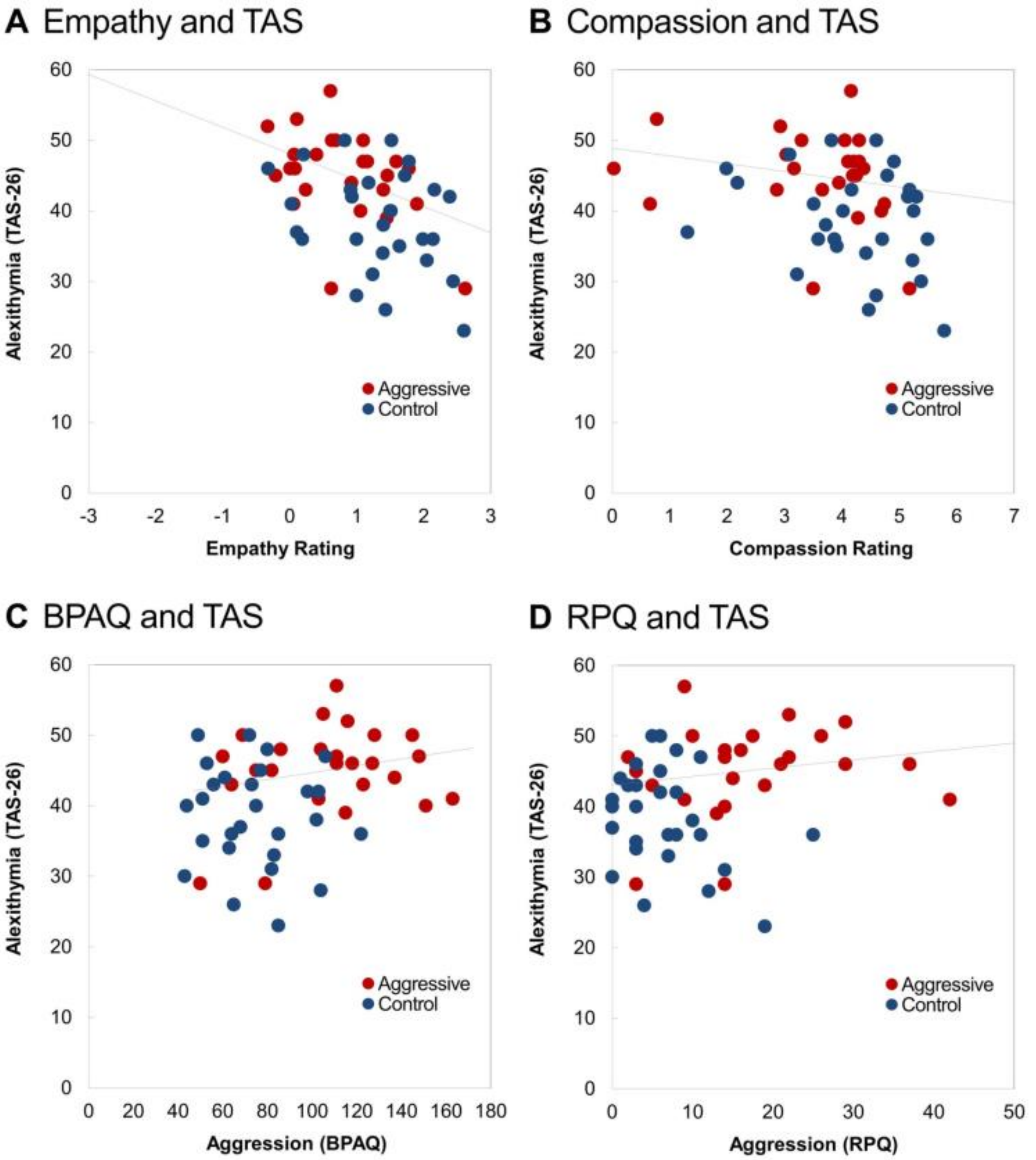

S7 Results of the mediation analysis with covariates: *IQ and years of education influence alexithymia in mediating empathic responding and compassion significantly.*

Covariate	*Model*	*Coefficient*	*SE*	*t*	*p*
	Alexithymia mediates reduced empathic responding				
	Model summary	R^2 = .1004, F(2.50) = 5.6940, p = .0208*			
none	Effect of Group affiliation (GA) on mediator	-6.4686	1.8870	-3.4279	.0012**
	Direct effect of mediator on empathic responding	-.0424	.0168	-2.513	.0150**
	Total effect of GA on empathic responding	.4988	.2126	2.3457	.0229**
	Direct effect of GA on empathic responding	.2248	2528	.8892	.3781
	Indirect effect of GA on empathic responding through mediator	effect = .2740 (SE = .1260), 95%CI: .0737 to .6027			
Note: significant mediation as path a, b, c are significant but not c'					
	Alexithymia mediates reduced empathic responding				
	Model summary	R^2 = .1133, F(2.50) = 3.1959, p = .0494*			
	Effect of Group affiliation (GA) on mediator	-5.398	2.0702	-2.6075	.0012**
IQ	Direct effect of mediator on empathic responding	-.0413	.015	-2.7473	.0084**
	Total effect of GA on empathic responding	.4103	.2338	1.7549	.0854
	Direct effect of GA on empathic responding	.1876	.2343	.8005	.4273
	Indirect effect of GA on empathic responding through mediator	effect = .2227 (SE = .1408), 95%CI: .0351 to .6264			
Note: no significant mediation (a, b path significant, but not c)					
	Alexithymia mediates reduced empathic responding				
	Model summary	R^2 = .1297, F(2.50) = 6.233, p = .0311**			
	Effect of Group affiliation (GA) on mediator	-5.0666	1.8628	-2.7199	.009**
**Years of Education**	Direct effect of mediator on empathic responding	-.04	.0157	-2.5503	.0139**
	Total effect of GA on empathic responding	.413	.2179	1.8952	.0639
	Direct effect of GA on empathic responding	.2101	.2216	.9481	.3477
	Indirect effect of GA on empathic responding through mediator	effect = .2029(SE = .1322), 95%CI: .0199 to .5568			
Note: no significant mediation (a, b path significant, but not c)					
	Alexithymia mediates reduced compassion				
	Model summary	R^2 = .0789, F(2.50) = 4.3664, p = .0417*			
	Effect of Group affiliation (GA) on mediator	-6.4686	1.8622	-.4736	.0011**
none	Direct effect of mediator on empathic responding	-.0424	.0243	-1.7400	.0880
	Total effect of GA on empathic responding	.6898	.3301	2.0896	.0417*
	Direct effect of GA on empathic responding	.4158	.3600	1.1550	.2536
	Indirect effect of GA on empathic responding through mediator	effect = .2740 (SE = .1602), 95%CI: .0251 to .6758			
Note: no significant mediation (a, c path significant, but not c)					
	Alexithymia mediates reduced compassion				
	Model summary	R^2 = .806, F(2.50) = 2.1918, p = .1223			
	Effect of Group affiliation (GA) on mediator	-5.398	2.0702	-2.6075	.0012*
IQ	Direct effect of mediator on empathic responding	-.0422	.0249	-1.6941	.0966
	Total effect of GA on empathic responding	.6392	.3716	1.72	.0916
	Direct effect of GA on empathic responding	.4112	.3889	1.0576	.2954
	Indirect effect of GA on empathic responding through mediator	effect = .2279 (SE = .1693). 95%CI: .0143 to .6526			
Note: no significant mediation (a path significant, but not b and c)					
	Alexithymia mediates reduced compassion				
	Model summary	R^2 = .0803, F(2.50) = 2.1817, p = .1235			
	Effect of Group affiliation (GA) on mediator	-5.0666	1.8628	-2.7199	.009**
**Years of Education**	Direct effect of mediator on empathic responding	-.045	.026	-1.7284	.0902
	Total effect of GA on empathic responding	.6605	.3497	1.8891	.0647
	Direct effect of GA on empathic responding	.4362	.3674	1.773	.2448
	Indirect effect of GA on empathic responding through mediator	effect = .2280 (SE = .1688), 95%CI: .0053 to .6953			
Note: no significant mediation (a path significant, but not b and c)					

*Note: *indicates statistical significant p-value: * $p \leq 0.05$, ** $p \leq 0.01$, *** $p \leq 0.001$*

S8 Intragroup correlations between IQ/years of education and EmpaToM measures: *No correlation between empathy measures and years of education or IQ nor between compassion measures and years of education or IQ were found, when within-group correlational analyses were carried out.*

		Aggressive		***Controls***	
		r	p value	r	p value
IQ	Empathy rating (EmpaToM)	.287	.155	-.140	.451
	Compassion rating (EmpaToM)	.004	.984	-.111	.551
Years of Education	Empathy rating (EmpaToM)	.174	.397	.063	.726
	Compassion rating (EmpaToM)	-.019	.926	.044	.806
